# The preconditioning of lithium promotes mesenchymal stem cell-based therapy for the degenerated intervertebral disc via upregulating cellular ROS

**DOI:** 10.1186/s13287-021-02306-9

**Published:** 2021-04-14

**Authors:** Zemin Zhu, Hongyuan Xing, Ruofu Tang, Shengjun Qian, Shaoqi He, Qiang Hu, Ning Zhang

**Affiliations:** 1 Department of Orthopedics, Changxing People’s Hospital, Changxing, China; 2 Department of Orthopaedics, 2nd Affiliated Hospital, School of Medicine, Zhejiang University, #88 Jiefang Road, Hangzhou, China; 3 Department of Orthopedics, Third Affiliated Hospital of Wenzhou Medical University, Wenzhou, China; 4 Department of Orthopedics, Pujiang Tianxian Orthopedic Hospital, Jinhua, China

**Keywords:** Lithium, Adipose-derived stem cell, Intervertebral disc degeneration, Precondition, Cell adaptation

## Abstract

**Abstract:**

Adipose-derived stem cell (ADSC) is one of the most widely used candidate cell for intervertebral disc (IVD) degeneration-related disease. However, the poor survival and low differentiation efficacy in stressed host microenvironment limit the therapeutic effects of ADSC-based therapy. The preconditioning has been found effective to boost the proliferation and the functioning of stem cells in varying pathological condition. Lithium is a common anti-depression drug and has been proved effective to enhance stem cell functioning. In this study, the effects of preconditioning using LiCl on the cellular behavior of ADSC was investigated, and specially in a degenerative IVD-like condition.

**Method:**

The cellular toxicity on rat ADSC was assessed by detecting lactate dehydrogenase (LDH) production after treatment with a varying concentration of lithium chloride (LiCl). The proliferative capacity of ADSC was determined by detecting Ki67 expression and the relative cell number of ADSC. Then, the preconditioned ADSC was challenged by a degenerative IVD-like condition. And the cell viability as well as the nucleus pulpous (NP) cell differentiation efficacy of preconditioned ADSC was evaluated by detecting the major marker expression and extracellular matrix (ECM) deposit. The therapeutic effects of preconditioned ADSC were evaluated using an IVD degeneration rat model, and the NP morphology and ECM content were assessed.

**Results:**

A concentration range of 1–10 mmol/L of LiCl was applied in the following study, since a higher concentration of LiCl causes a major cell death (about 40%). The relative cell number was similar between preconditioned groups and the control group after preconditioning. The Ki67 expression was elevated after preconditioning. Consistently, the preconditioned ADSC showed stronger proliferation capacity. Besides, the preconditioned groups exhibit higher expression of NP markers than the control group after NP cell induction. Moreover, the preconditioning of LiCl reduced the cell death and promoted ECM deposits, when challenged with a degenerative IVD-like culture. Mechanically, the preconditioning of LiCl induced an increased cellular reactive oxidative species (ROS) level and activation of ERK1/2, which was found closely related to the enhanced cell survival and ECM deposits after preconditioning. The treatment with preconditioned ADSC showed better therapeutic effects than control ADSC transplantation, with better NP preservation and ECM deposits.

**Conclusion:**

These results suggest that the preconditioning with a medium level of LiCl boosts the cell proliferation and differentiation efficacy under a normal or hostile culture condition via the activation of cellular ROS/ERK axis. It is a promising pre-treatment of ADSC to promote the cell functioning and the following regenerative capacity, with superior therapeutic effects than untreated ADSC transplantation.

## Introduction

Low back pain (LBP) is one of the major causes of disability in the elderly [1]. The intervertebral disc degeneration (IVDD) is considered the main cause of LBP [2]. An alteration in the niche of nucleus pulpous (NP) cells results in cell death and the matrix imbalance of NP, with the denaturation of type II collagen (Col II) and the loss of glycosaminoglycan. Unfortunately, there is no ideal treatment so far. The current treatment includes conservative and surgical therapies, which cannot stop the degeneration process of IVD, nor reverse the degeneration [3].

Cell-based therapy has shown a great potential in the treatment of multiple diseases or pathological processes [4]. Because of the pluripotent property and an easy access, adipose-derived stem cell (ADSC) has attracted many attentions in bioengineering medicine [5, 6]. However, due to the harsh condition in degenerative intervertebral disc, the therapeutic effects of cell-based therapy is adversely limited, due to the poor survival and the impaired cell viability. Chan et al. reported only 20% of cells survived for over 7 days after transplantation into simulated-physiological conditions of cryopreservation IVD [7, 8]. Specifically, previous researchers have proven that the acidic condition as well as high osmotic condition both impair the cell viability and proliferation, as well as the extracellular matrix (ECM) deposits [1, 9–12]. The urgent need of candidate cells that could adapt to IVD condition requires further study.

To promote the adaptation and the functioning of the transplanted cells in pathological condition, the preconditioning treatment has been developed and proved feasible [13, 14], mainly including some chemical or biological factors [15, 16]. Due to the limited space in the degenerated disc, the preconditioned cell shows advantages of higher feasibility and integration, with lower chance to cause unexpected effects by implanted materials. Recently, the preconditioning using lithium has been found beneficial in cell-based therapy against disease models in brain, bone, and heart [17–21]. Lithium could promote the proliferation of bone marrow-derived mesenchymal stem cell (BMSC) via a glycogen synthase kinase (GSK) 3β-dependent β-catenin/Wnt pathway [22]. Besides, the preconditioning with lithium has been found to reduce cell apoptosis by inducing autophagy [23]. Above studies suggest that the preconditioning of lithium would be a promising method to boost the cellular adaptation in a stressed microenvironment. While the specific influence of preconditioning with lithium to cellular adaptation of ADSC in the IVDD-like condition remains elusive.

In this regard, we designed this study to investigate the potential value of the preconditioning with lithium for an ADSC-based IVDD treatment. The cellular adaptation including cell proliferation and differentiation in normal or the IVDD-like condition were evaluated, and the therapeutic effects of preconditioned ADSC was also assessed using an IVDD rat model.

## Material and methods

### Cell culture and treatment

The Sprague-Dawley (SD) rat ADSCs of passage 2 were purchased from Cyagen Bioscience (China). Cells were cultured using SD rat ADSC basal medium supplemented with 10% fetal bovine serum (FBS), 1% glutamine, and 1% penicillin-streptomycin. The medium was replaced twice every week, and cells at passages 4 were used in subsequent experiments. The ADSC microsphere was also prepared. Every 1 mL cell suspension containing 3 × 10^5^ cells was added to 15 mL centrifuge tube and centrifuged at 300 g/min at 4 °C for 3 min. The microsphere forms after incubation overnight.

In regard to the preconditioning treatment, ADSCs were implanted on 6-well plates at a density of 3 × 10^5^ cells/well and maintained overnight for adhesion. LiCl aqueous solution was added into basal medium at a final concentration of 0, 1, 4, 10, and 20 mmol/L. The preconditioned ADSC was also prepared for transplantation in vivo. Specifically, each 1 × 10^6^ cells were resuspended using basal medium and kept on ice till use.

The cellular ROS production was blocked using apocynin (APO, 5 μM). Cells were maintained in LiCl-containing medium with or without APO for 48 h and washed with PBS.

The NP-like cell differentiation medium was built using the high glucose Dulbecco’s modified Eagle’s medium (DMEM), supplemented with 10% FBS, insulin-transferrin-selenium (ITS), dexamethasone (40 ng/mL), 50 nM ascorbic acid-2-phosphate, TGF-β3 (10 ng/ mL), and IGF-1 (10 ng/mL).

The degenerative IVD-like condition was built according to previous reports [24, 25]. Briefly, the hyperosmotic condition (485 mOsm) was achieved by an addition of 2% potassium chloride (0.1 M) and sodium chloride (5 M), and the acidic condition (pH = 6.8) was built with the 0.2% hydrogen chloride (1 M), which were added into the low glucose Dulbecco’s modified Eagle’s medium (DMEM) [7, 24, 26].

### Cellular proliferation assay

The cell viability was determined using a Trypan Blue staining reagent. The proliferation ability of ADSC after preconditioning was determined by counting the cell number and the lactate dehydrogenase (LDH) assay. The relative cell number was monitored using the cell counting kit (CCK8, dojindo) before treatment, after treatment, and at 2 days post-treatment. Briefly, cells were implanted in 96-well plate at a density of 1 × 10^4^ cells/well. The supernatant was discarded, then the cells were incubated with 5% CCK8 in high glucose DMEM for 2 h. The supernatant was collected and the absorption at 490 nm was detected using a spectrophotometer. The LDH assay was performed according to the instruction of the manufacturer. The culture medium was collected after preconditioning and 2 days after transplantation into the degenerative condition. The LDH content was determined by detecting the absorption at 440 nm was detected using spectrophotometer.

### Immunofluorescent staining

The cell proliferation ability was also determined by detecting the Ki67 level after preconditioning. Briefly, cells cultured in 12-well plate were washed using cold PBS and immobilized using 4% paraformaldehyde for 30 min. Then, cells were permeabilized using PBS-Triton (0.5%) for 15 min. After these, cells were blocked using 5% bovine serum albumin (BSA) at RT for 1 h, and incubated overnight at 4 °C with Ki67 (ab15580) primary antibody.

After washing three times using PBS, the cells were incubated with secondary antibodies conjugated to the Alexa Fluor fluorescent dyes (ab150078). Subsequently washed with PBS, cells were stained with 4, 6-diamidino-2-phenylindole (DAPI, Sigma-Aldrich) for 5 min and washed with PBS for observation. Images were captured using a fluorescent microscope.

### Western blot analysis

Western blot analysis was performed to determine the protein content of major NP cell markers. Briefly, cells were washed three times with cold PBS and lysed at 4 °C using RIPA buffer (Beyotime) for 30 min. Protein contents were measured using the BCA protein assay. For each sample, 30 μg total proteins were loaded and separated by sodium dodecyl sulfate polyacrylamide gel electrophoresis (SDS-PAGE) and transferred onto an immobilon polyvinylidene difluoride (PVDF) membrane (Millipore Billerica). The membranes were blocked using 10% bovine serum albumin (BSA) and incubated with anti-GAPDH antibody (CST#5174), anti-type II collagen antibody (CST#13120), anti-aggrecan antibody (CST#13120) and anti-Krt19 antibody (CST#12434), anti-SOX9 antibody (CST#13120), anti-GSK-3b, anti-MMP13, anti-ADAMTS5, anti-ERK1/2, and anti-pERK1/2 overnight at 4 °C. Then, the blots were rinsed and incubated with HRP-labeled secondary antibody (Bioker, China) for 2 h at room temperature (RT). The protein bands were visualized using a Bio-Rad imaging system, and the relative protein intensity of the control was calculated using “Quantity One” software.

### Gene expression analysis

RT-PCR was performed to assess the mRNA level of the essential markers in the preconditioned ADSC under normal condition or IVDD condition after differentiation. Briefly, RNAiso reagent was used to extract total RNA. And the PrimeScriptTM Reagent Kit was used for reverse transcription. Quantitative polymerase chain reaction (qPCR) was performed using SYBR Green (Takara) according to the instruction of the manufacturer. Primers were synthesized by Sangon Biotech (China), and the sequences are listed Table 1.

**Table 1:** Primers used in quantitative PCR

	Primer, 5′–3′	
Gene	Forward	Reverse
*18S*	GAATTCCCAGTAAGTGCGGGTCATA	CGAGGGCCTCACTAAACCATC
*Acan*	CTAGCTGCTTAGCAGGGATAACG	GATGACCCGCAGAGTCACAAAG
*Col II*	CTGGTGGAGCAGCAAGAGC	GTGGACAGTAGACGGAGGAAAG
*Sox 9*	AGGAAGCTGGCAGACCAGTACC	GGGTCTCTTCTCGCTCTCGTTCA

### Alcian blue staining

The ECM deposit was also determined by the Alcian blue staining. Briefly, after differentiation, cells were washed with PBS, then fixed with 4% paraformaldehyde for 15 min. The cells were stained using Alcian blue solution for 30 min and washed twice with PBS before observation under microscope.

### Cellular ROS level detection

The cellular ROS production of ADSC was detected using 2′, 7′-dichlorofluorescein diacetates (DCFH-DA, Sigma-Aldrich) as previously reported [27]. Cells were washed with PBS after treatment, then the DCFH-DA was loaded at a concentration of 20 mmol/L in DMEM medium for 30 min, and the cells were washed thrice with PBS. The fluorescent image was taken with the fluorescence microscope.

### Animal surgery

SD rats (150 g) were purchased from the Shanghai SLAC Animal Research Center. All procedures in this study was approved by the ethic Committee of Zhejiang Chinese Medicine University Laboratory Animal Research Center. The needle puncture-induced model was applied in here. Twenty rats were divided into four groups: normal control (without needle puncture or treatment), degeneration group (needle puncture with PBS treatment), ADSC-treated group (needle puncture with ADSC transplantation), and preconditioned ADSC (needle puncture with preconditioned ADSC transplantation). Rats were anesthetized using 2% pentobarbital sodium intraperitoneally, and a sterile 20-gauge needle was inserted just through the annulus fibrosus (AF) into the middle of the NP, and rotated for 360°, then held for 30 s. The treatment was applied at 2 weeks post puncture, and the total injection volume was about 2 μL.

### Histological and biochemical analysis

The rats were sacrificed by euthanasia at 3 month after treatment. The specimens were harvested and fixed using 4% PFA, embedded in paraffin, and sectioned at 5 μm thickness. The slices were stained with hematoxylin and eosin (H&E) or safranin O-fast green (S-O).

For immunohistochemical (IHC) staining, the slices were deparaffinized in xylene and rehydrated, then treated with 3% H_2_O_2_ for 10 min, then blocked with 5% bovine serum albumin (BSA) for 30 min at room temperature. After that, the slices were incubated with diluted primary antibody at 4 °C overnight. After washes with PBS, the slices were incubated at 37 °C for 30 min with biotin-labeled secondary antibody, and the staining was detected by the streptavidin-biotin complex (SABC) method.

The biochemical analysis was performed according to previous report [3]. Briefly, the NP samples were harvested and frozen at − 80 °C and lyophilized for 24 h. The samples were weighted and recorded, then digested with papain (Sigma) for 18 h at 60 °C. The contents of sulfated glycosaminoglycans (sGAG) and hydroxyproline of each group were detected using the DMMB assay and Hydroxyproline Assay Kit (Jiancheng Bioengineering Institute, China), respectively. The results were normalized with dry weight of NP sample.

### Statistical analysis

Results are expressed as the mean ± standard deviation for at least three independent experiments. Data were analyzed using one-way analysis of variance (ANOVA, non-parametric test) followed by Dunn’s post hoc test for multiple comparisons (SPSS 20). A value of *P* < 0.05 was considered to indicate statistical significance.

## Results

### Cellular toxicity of Li

**Fig. 1:**
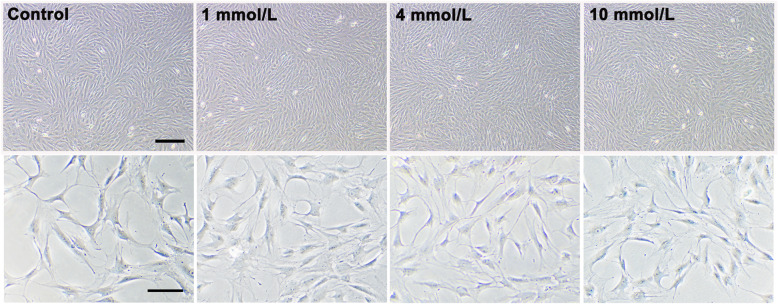
Morphology characterization after the preconditioning with LiCl. No obvious morphological changes observed between each group. Scale bar = 100 μm (up), 10 μm (down)

About 40% cells were found dead after 2-day treatment with 20 mmol/L LiCl; thus, lower concentration was applied in subsequent experiments. The cell morphology was also monitored, with similar cell shape between each group after preconditioning (Fig. 1). The cellular viability was determined using Trypan Blue staining reagent. The result indicated no significant alteration on the live cell ratio after preconditioning (the data did not show). Then the cell death was also determined using the LDH assay. And the result showed similar LDH level among the groups with 0, 1, 4, and 10 mmol/L LiCl in basal culture medium (data provided in Fig. 5b). These data indicated minimal cellular toxicity of LiCl to ADSC when the concentration was under 10 mmol/L.

### Preconditioning of LiCl promotes the proliferation of ADSC

**Fig. 2:**
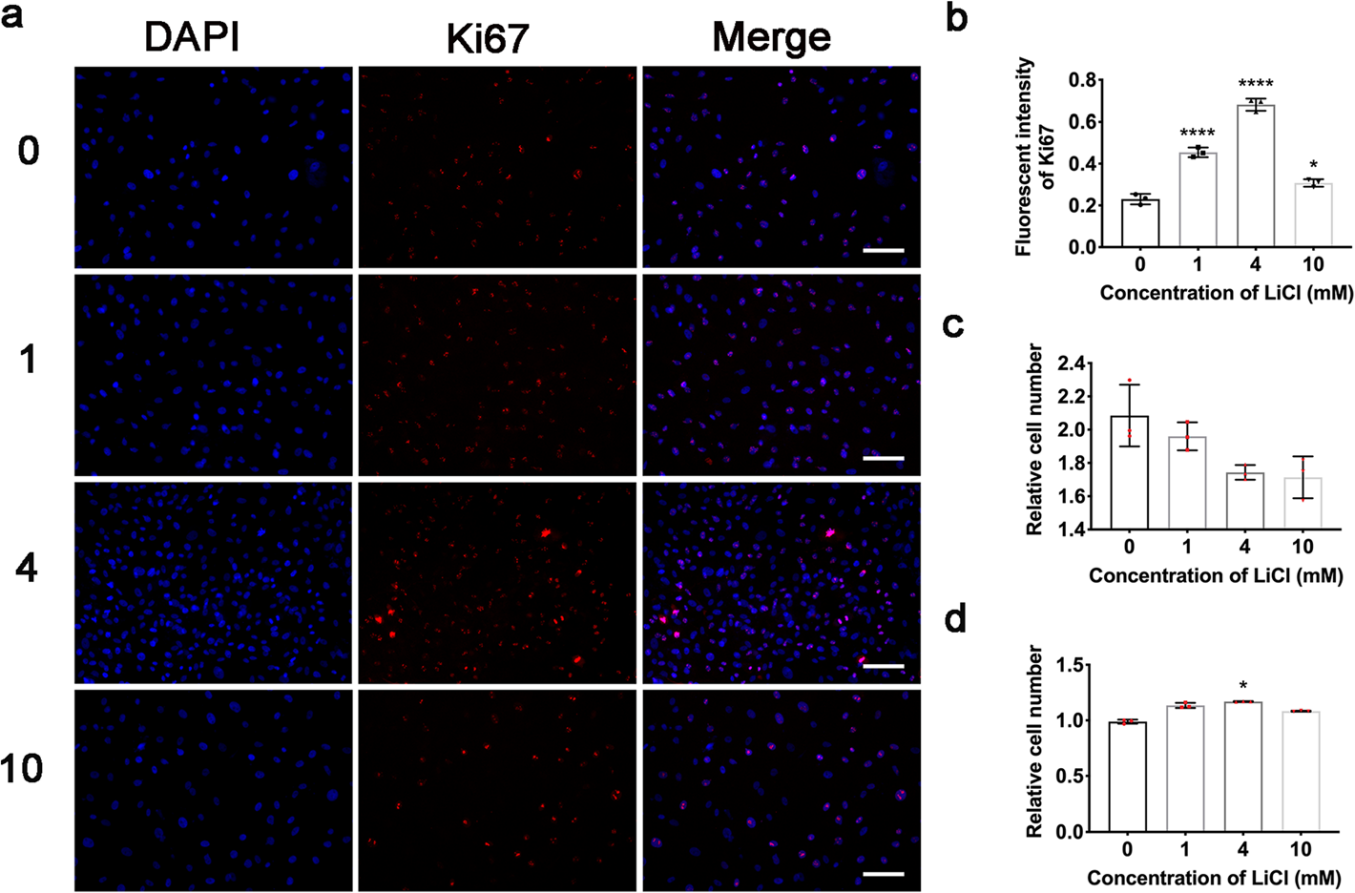
Preconditioning using LiCl promotes ADSC proliferation. **a** Immunofluorescent staining of Ki67 after preconditioning, scale bar = 200 μm. **b** Quantitative analysis of Ki67 content. **c** Relative cell number of ADSC after preconditioning. **d** Two-day-post preconditioning. Error bars depict Mean ± SD. (****P < 0.0001, ***P < 0.001, **P < 0.01, *P < 0.05)

Then, the cellular proliferation capacity was assessed by cell number counting and the Ki67 level detection after the preconditioning of LiCl. The content of Ki67 was found increased after preconditioning by immunofluorescent staining (Fig. 2a, b). And the medium group (4 mmol/L) resulted in a highest promotion (*P* = 0.0134) of Ki67, while other groups showed no significant difference compared to control.

The CCK8 kit was used to monitor the relative cell number. All preconditioned groups showed equivalent cell number with control (Fig. 2c), while the preconditioned groups with 4 mmol/L were found with an enhanced proliferation capacity than the control after the preconditioning (Fig. 2d) (*P* = 0.0134). These results indicated a boost on the proliferation ability of ADSC by the preconditioning with a medium concentration of LiCl.

### Preconditioning of LiCl enhances the NP-like cell differentiation

**Fig. 3:**
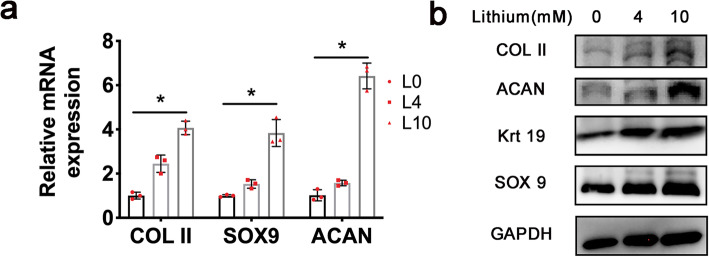
Preconditioning of LiCl promotes the NP cell phenotype differentiation efficacy. **a** Relative mRNA expression of NP cell markers after preconditioning. **b** Protein level of NP cell markers after preconditioning. Error bars depict Mean ± SD. (*P < 0.05)

In addition to the proliferation ability, the NP-like cell differentiation potency was further evaluated to determine the influence of preconditioning with LiCl. Cells were transplanted to NP cell differentiation medium after preconditioning. The mRNA level of major NP cell markers was assessed, including Col II, Aggrecan (ACAN), and SOX9. The result showed no significant changes on the mRNA level of these markers after the preconditioning between each group (the result did not show). Notably, the level of these markers were found significantly increased in the 10 mmol/L preconditioned group than the control after a subsequent NP cell differentiation for 2 weeks (Fig. 3a) (*P* = 0.0219).

To verify the results, the protein level of these markers was also detected, which was consistent with the mRNA expression pattern (Fig. 3b). Specifically, the content of ACAN and SOX9 were higher in the 10 mmol/L group than other groups. The expression of Col II (*P* < 0.0001) and Krt19 (*P* < 0.001) were found increased in the 4 mmol/L group and the 10 mmol/L group, when compared to the control. These results suggested that the preconditioning could also effectively boost the NP-like cell induction efficacy of ADSC, compared to naïve ADSC.

### Preconditioning of LiCl promotes cellular adaptation of ADSC to degenerative IVD-like condition

**Fig. 4:**
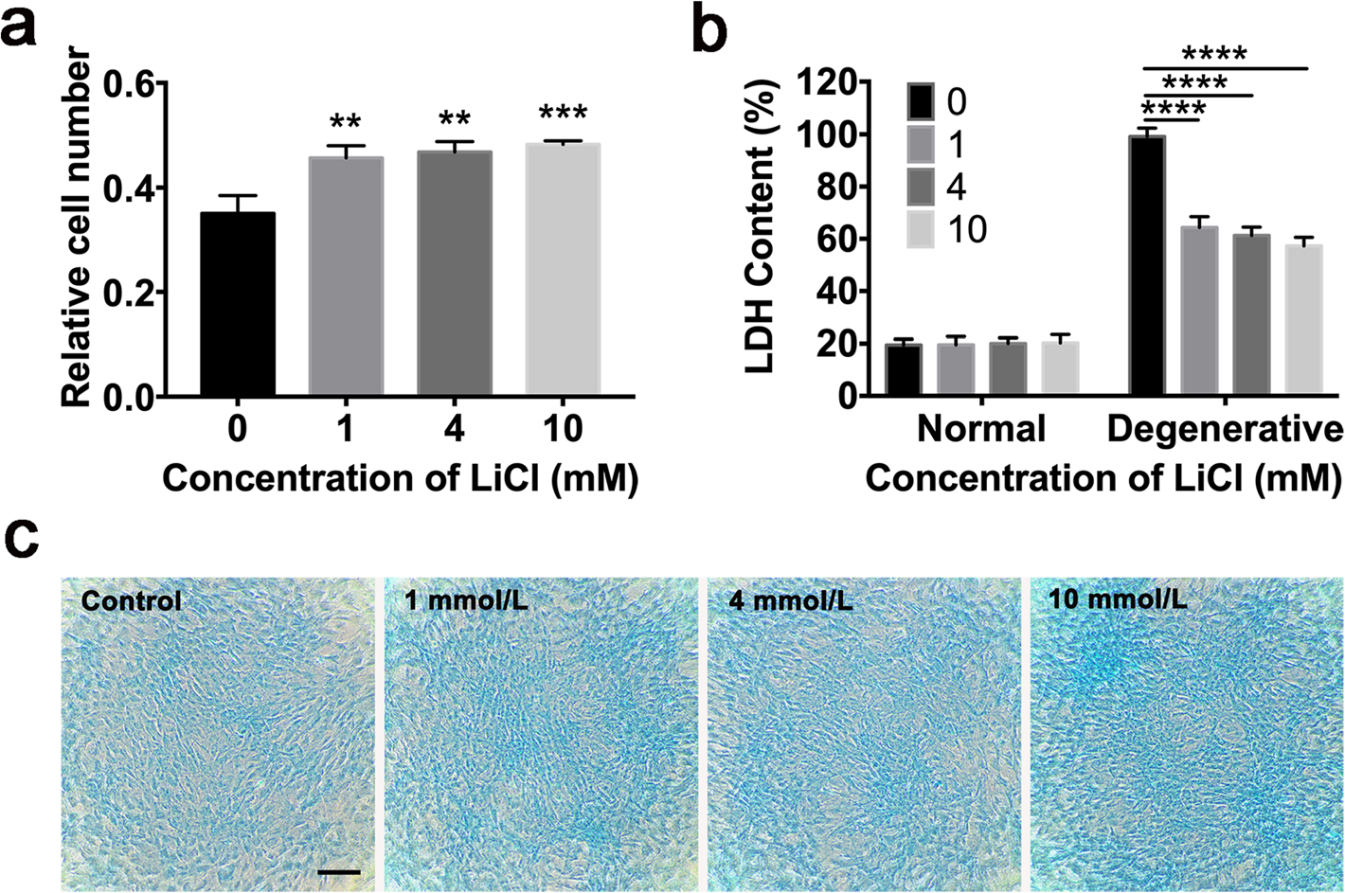
Preconditioning using LiCl promotes cell survival and ECM deposit in the degenerative IVD-like condition. **a** Relative cell number at 2 days post-transplantation. **b** Content of LDH of ADSC in normal condition or degenerative IVD-like condition. **c** The ECM content was determined using Alcian Blue staining. Scale bar = 100 μm. Error bars depict Mean ± SD. (****P < 0.0001, ***P < 0.001, **P < 0.01)

The above data suggests that the preconditioning of LiCl can boost the proliferation ability and the NP-like cell induction efficacy of ADSC in normal culture condition. The hostile microenvironment in degenerated IVD is one of the major obstacles for ADSC functioning in vivo. To test the effects of preconditioning on the cellular adaptation to degenerative IVD-like condition, the treated cells were then challenged with the degenerative IVD-like condition, characterized by hyperosmotic pressure, acidic environment, and a lower nutrition, according to previous reports [24]. The control group showed a sharp decrease of cell number, while those preconditioned groups did not. The result of CCK8 showed an over 64% cell loss in the control group, and with about 10%, 12%, and 13% more cells survived in preconditioned groups, respectively (Fig. 4a). In addition, the cell death was confirmed using the LDH assay, which was consistent with the result of CCK8, suggesting a reduced cell death in all three preconditioned groups in degenerative IVD-like condition (Fig. 4b) (*P* < 0.0001).

**Fig. 5:**
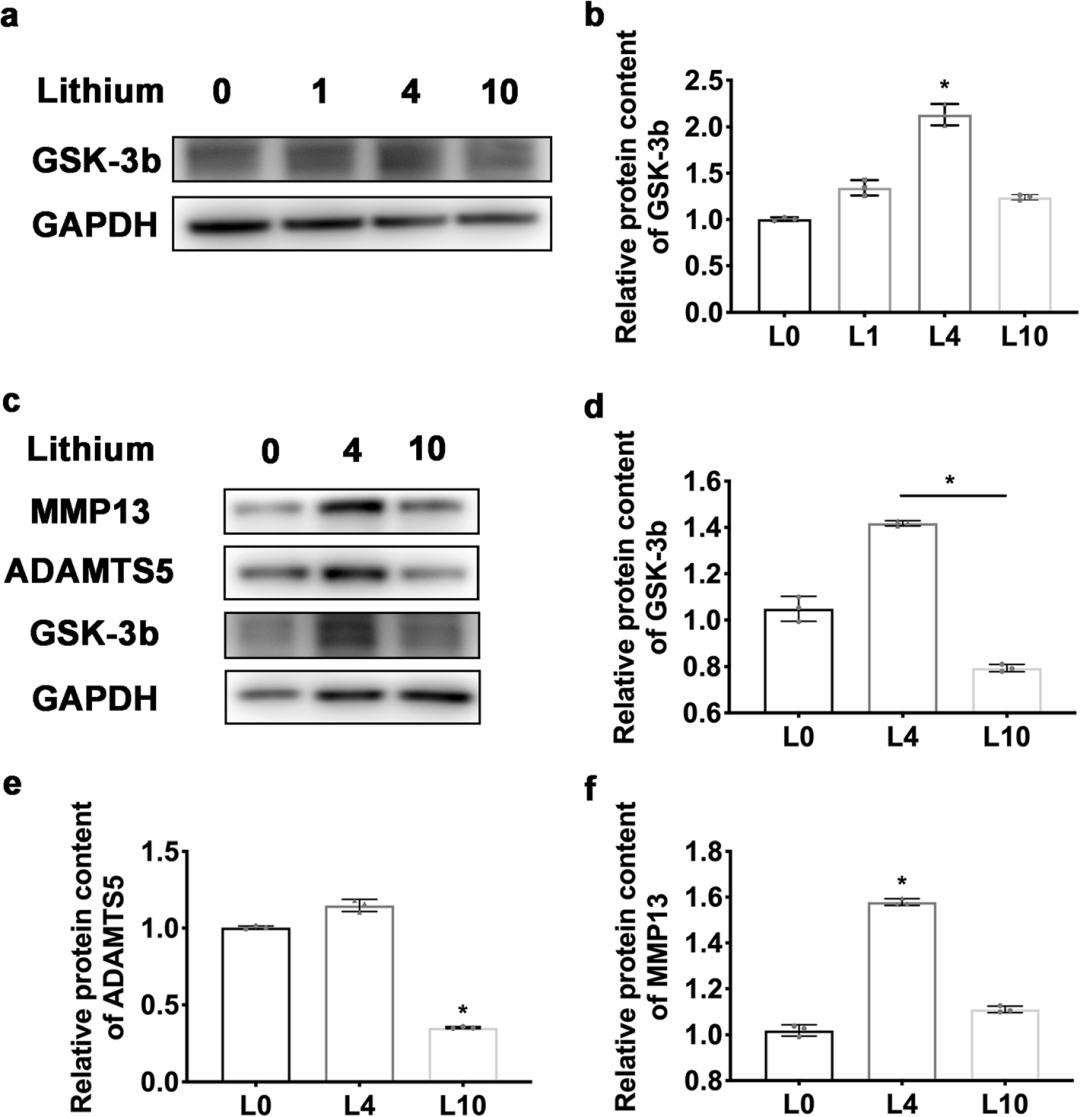
The preconditioning using LiCl did not elevate the expression of GSK-3b and suppressed catabolism activity. **a**, **b** The expression of GSK-3b after preconditioning and **c**, **d** after NP differentiation. **e, f** The protein content of MMP13 and ADAMTS5 after differentiation. Error bars depict Mean ± SD. (*P < 0.05)

Besides, the preconditioned ADSCs were cultured in NP cell induction medium and challenged by a degenerative IVD-like condition. After 2 weeks, the ECM deposit was detected by Alcian blue staining. As a result, the preconditioned ADSCs exhibited more ECM deposit than control ADSC (Fig. 4c). The expression matrix metalloproteinase 13 (MMP13) and ADAMTS5 were also tested. The results showed a reduction on ADAMTS5 in 10 mmol/L preconditioned group (*P* = 0.0219). Also there was no increase of MMP13 in 10 mmol/L preconditioned group, though an increase of MMP13 in 4 mmol/L preconditioned group was found (Fig. 5c, e, f) (*P* = 0.0219). These results suggested an enhanced adaptation of ADSC to the degenerative IVD-like condition after preconditioning with LiCl, with a reduced cell death and enhanced ECM synthesis ability, as well as a reduced matrix catabolism, especially when preconditioned using 10 mmol/L LiCl.

Before our assessment of the NP-regenerative effects of LiCl-preconditioned ADSCs in animal model, the inhibitory effects of LiCl preconditioning on GSK-3b was assessed, due to the potentially subsequent activation on Wnt/β-catenin pathway after GSK-3b inhibition, which could cause negative effects on NP regeneration. The expression of GSK-3b by ADSCs was evaluated after preconditioning and after differentiation (Fig. 5a–d). The results showed no significant reduction of GSK-3b in preconditioned ADSC when compared to untreated ADSC. Notably, the medium level of preconditioning even increased the GSK-3b level (*P* = 0.0134). The content of β-catenin was also tested (data not shown), but the level was not elevated. These data suggested a promotion of adaptation capacity of ADSCs to the degenerative IVD condition after LiCl preconditioning, without causing significant GSK-3b inhibition nor Wnt/β-catenin pathway activation.

### Therapeutic effects of preconditioned ADSC in vivo

**Fig. 6:**
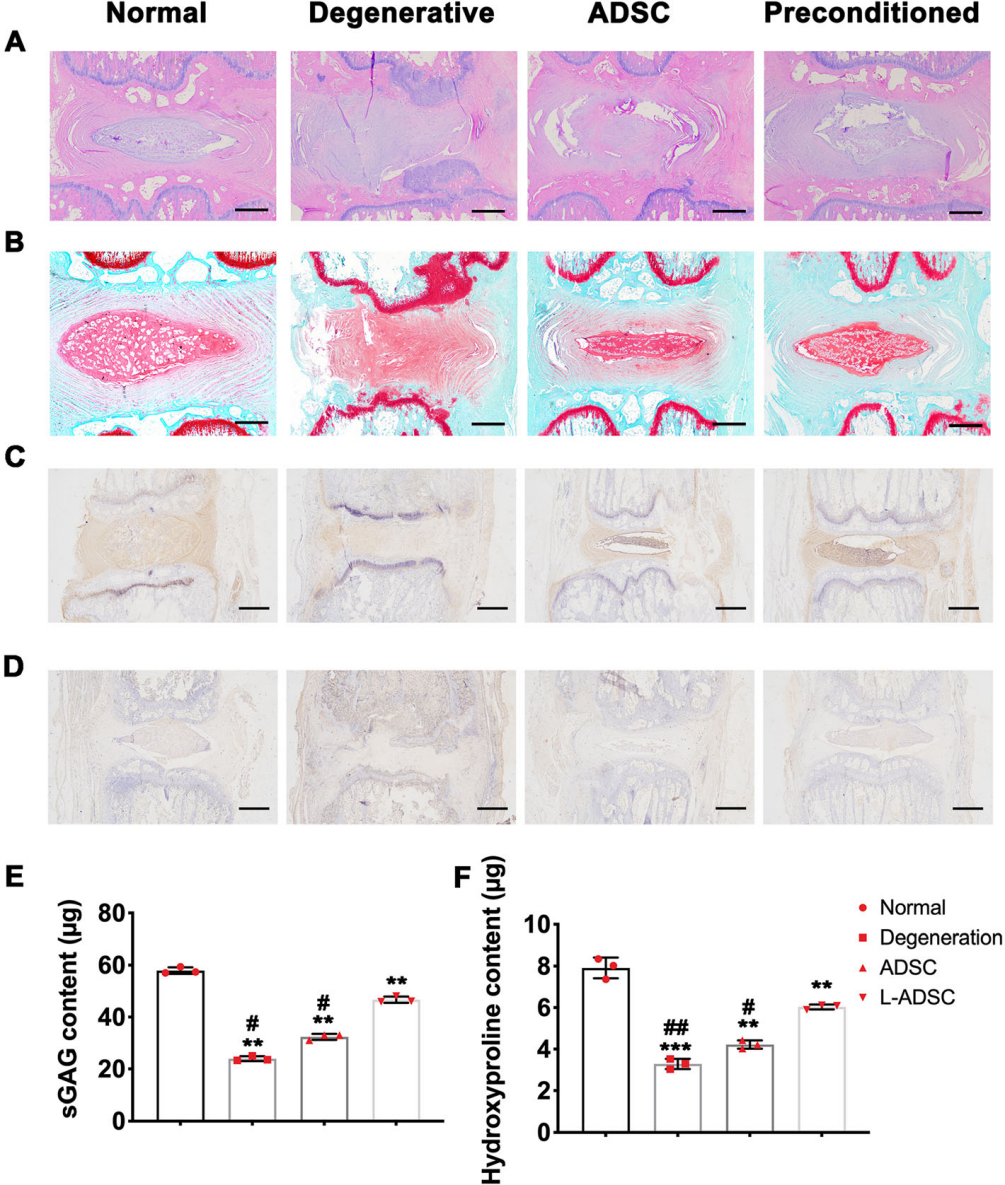
LiCl-preconditioned ADSC exhibited NP-regenerative effects. **a** H&E staining, **b** S-O staining, and IHC staining of **c** Col II and **d** aggrecan of IVD tissue at 3 months after treatment. **e** The sGAG content normalized to dry NP weight. **f** Hydroxyproline content normalized to dry NP weight. Error bars depict Mean ± SD. **P < 0.001, *P < 0.05, compared to Normal control; **##***P* < 0.01, **#***P* < 0.05, compared to LiCl-preconditioned ADSC (L-ADSC)

Assessed after 3 months after treatment, the therapeutic effects of LiCl-preconditioned ADSCs were assessed with histological and biochemical methods. The well-organized cells and ECM was revealed in normal control group by H&E staining, while the NP structure was destroyed in degeneration group, with disappeared lamellar structure of AF and hyperplastic endplate cartilage. The preconditioned ADSC-treated group exhibited better preserved NP tissue than ADSC-treated control and degeneration group. Besides, the S-O staining showed remarkably reduced ECM content in degeneration group, when compared to the normal control. And the preconditioned ADSC-treated group was found with more ECM deposits than the ADSC-treated control, though still less than normal control (Fig. 6a, b).

The distribution of aggrecan and Col II in NP tissues was also determined using IHC staining (Fig. 6c, d). At 3 months after treatment, the degeneration group was found with little positive staining NP tissue. The aggrecan content were distinctly found in NP tissues in normal group and preconditioned ADSC-treated group. Fewer NP tissue was found with aggrecan staining in ADSC-treated control, when compared to preconditioned ADSC-treated group. The Col II staining showed a similar pattern that the preconditioned ADSC-treated group exhibited higher level of Col II content in NP tissue than ADSC-treated control, though still decreased than normal group.

The content of sGAG in NP tissues was also measured by the DMMB assay. The result revealed an increased sGAG content in preconditioned ADSC-treated rat, when compared to degeneration control and ADSC-treated control (Fig. 6e). The hydroxyproline content was also detected to determine the Col II content in NP tissue. The result revealed an increased Col II content after treatment with preconditioned ADSC after degeneration (Fig. 6f).

### The effects of preconditioning depends on the activation of cellular ROS/ERK axis

**Fig. 7:**
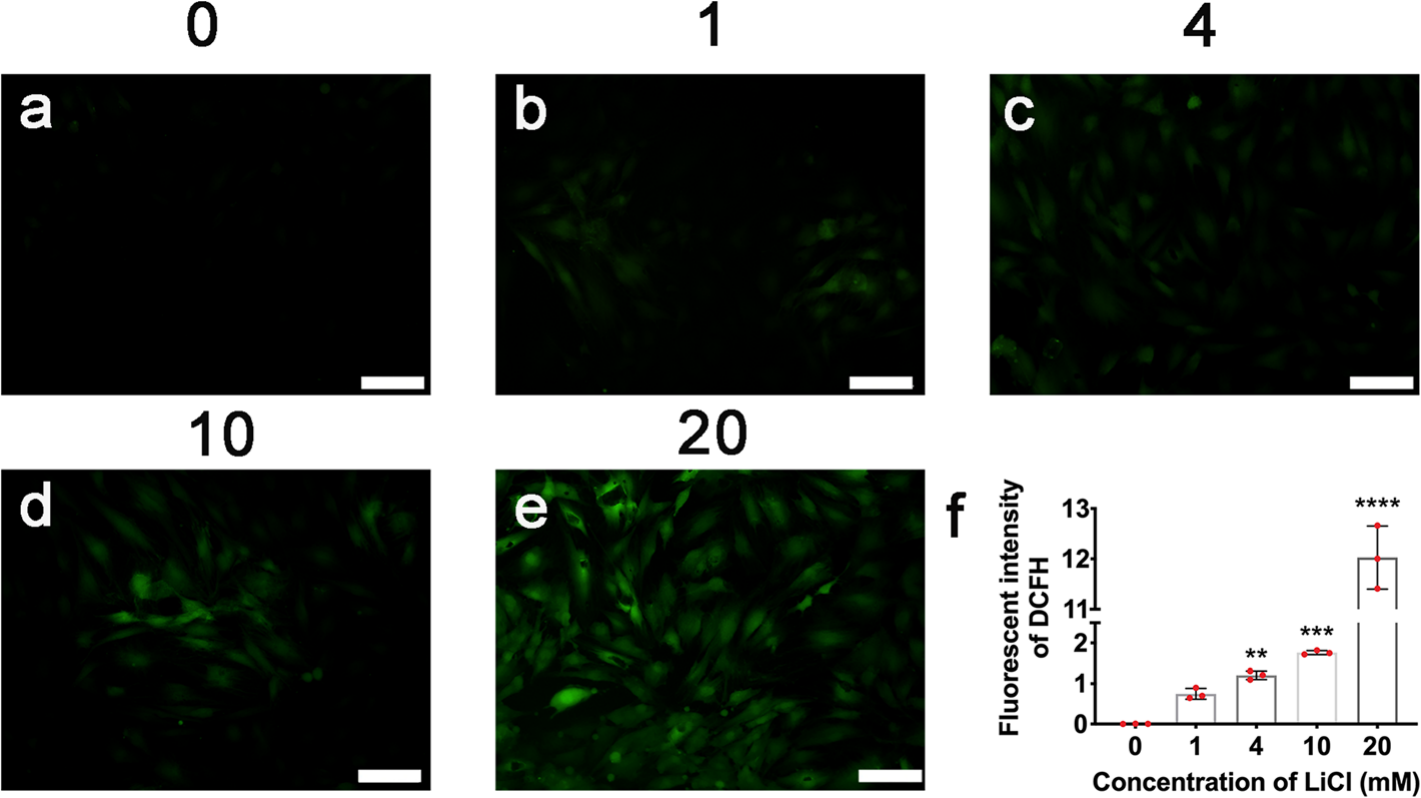
Preconditioning of LiCl increased cellular ROS level in ADSC. **a**–**e** The cellular ROS after preconditioning with LiCl was detected using DCFH-DA, scale bar = 100 μm. **f** Quantitative analyses of cellular ROS level. Error bars depict Mean ± SD. (****P < 0.0001, ***P < 0.001, **P < 0.01. NS, nonsignificant)

To investigate the potential mechanism of the promotion effects by LiCl preconditioning, the cellular ROS level was monitored as the ROS played key role in stem cell fate and function. The results showed a significantly increased ROS level in ADSC after the preconditioning of LiCl of 4–20 mmol/L (Fig. 7a, b).

**Fig. 8:**
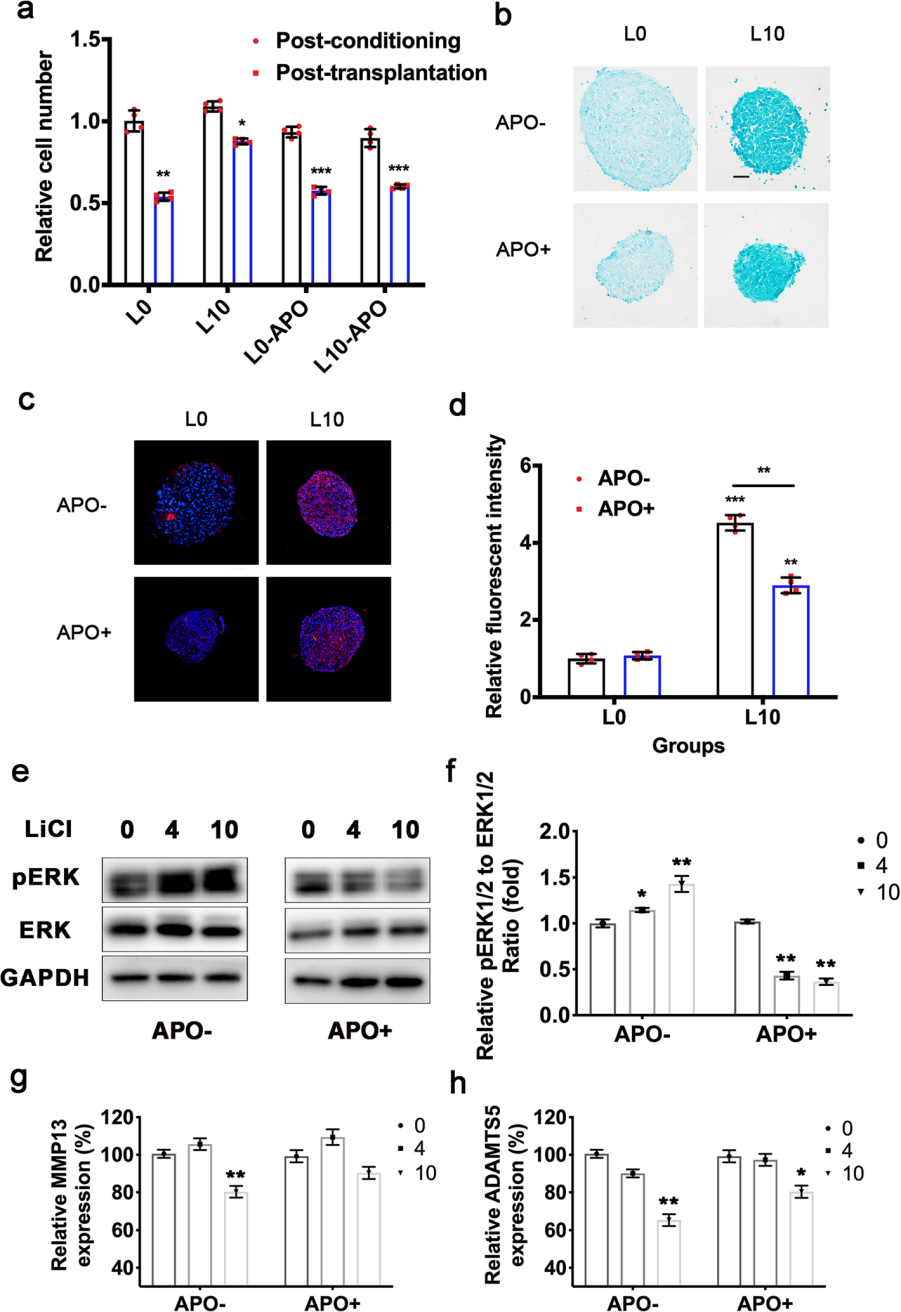
Prevention of ROS generation attenuated the effects of LiCl preconditioning to ADSC. **a** Relative cell number of ADSC microsphere. **b** The ECM content of ADSC microspheres determined using Alcian Blue staining, scale bar = 50 μm. **c** The immunofluorescent staining of aggrecan in ADSC microspheres, scale bar = 50 μm. **d** Quantitative analyses of aggrecan content. **e, f** ERK1/2 and pERK1/2 expression of preconditioned ADSC with or without APO treatment. **g, h** the mRNA expression of MMP13 and ADAMTS5 of preconditioned ADSC with or without APO treatment. Error bars depict Mean ± SD. (***P < 0.001, **P < 0.01, *P < 0.05)

To investigate the relationship between the elevated cellular ROS and the promotion of the cellular survival and the ECM deposits, ADSC microspheres were prepared and preconditioned with LiCl (10 mmol/L), with (APO+) or without APO (APO−), before NP induction. The cell number was similar between the preconditioned groups, while the APO+ LiCl-preconditioned group exhibited poor survival ability in degenerated IVD-like condition than the APO− LiCl-preconditioned group. (Fig. 8a) The result indicated the APO attenuated the promotion on the cellular survival by LiCl preconditioning.

The ECM deposits with or without APO preconditioning was also assessed after NP induction in degenerated IVD-like condition. The APO+ LiCl-preconditioned group was found with less ECM deposits than the APO− group, as revealed by the weaker staining intensity of Alcian blue and the immunofluorescent intensity of aggrecan (Fig. 8b–d).

To further investigate the underlying mechanisms of the LiCl-induced ROS-dependent behavior, the MAP kinase ERK1/2 axis was detected, since ROS is an important mediator of MAP kinase activation [28]. The results showed an elevated pERK1/2-ERK1/2 ratio after LiCl preconditioning. Besides, the prevention on the ROS generation during preconditioning, with APO, attenuated the activation of ERK1/2 (Fig. 8e, f). Accordingly, the suppression effect of LiCl preconditioning on MMP13 and ADAMTS5 was also attenuated (Fig. 8g, h).

Taken together, our data suggested a promotion on the cellular survival and ECM deposits, as well as a suppressed catabolism activity in ADSC under IVD condition after LiCl preconditioning, which were found dependent on the activation of ROS/ERK axis.

## Discussion

The cellular adaptation to a stressed host condition is essential for a successful therapy. NP cells have been found with superior tolerance to the IVD condition, including the survival and ECM synthesis ability, rather than naïve MSC. In this study, we investigated the influences of the preconditioning with LiCl on the cellular behavior of ADSC under normal culture condition, and the adaptation of the preconditioned ADSC to a degenerative IVD-like condition via a series of experiments. And the results suggested that the preconditioning of LiCl boosted the proliferation ability and promoted the cellular adaptation of ADSC to the degenerative IVD-like condition, which reduced the cell death effectively and benefited the ECM deposit of ADSC in vitro and in vivo. What is more, these effects were found closely related to the elevated cellular ROS and activation of ERK1/2 during LiCl preconditioning. The application of ROS scavenger during preconditioning significantly attenuated the promotion effects on the cellular adaptation of ADSCs in IVD condition.

The tissue microenvironment is composed of the biological condition and the physicomechanical and chemical condition, which affect almost every cellular activity via affecting transcriptional processes or major metabolic processes, etc.. Accordingly, various strategies have been built up to regulate cellular behaviors with biological factors, such as cytokines, or to change the physicomechanical property, or to change the content of specific components in milieus [29–31]. Compared to biological methods, the treatment using physicomechanical or inorganic elements is much easier to deliver and maintain, since the modulatory efficacy is believed to turn out more stable. For example, the iron balance theory has been widely applied in anti-cancer therapy by fascinating ferroptosis of cancer cells [32, 33]. Selenium is also found essential for the synthesis of selenoprotein GPX4, which is dispensable for normal embryogenesis [34]. Lithium has been used to treat manic depression for over 100 years. Besides, lithium has also been found to paradoxically reduce lymphocyte production, but enhance their function [35, 36]. Recent studies have also identified that the promotion of cellular function of MSC occurs after preconditioning with LiCl, which is also observed in this study, suggesting the promisingly therapeutic role in cell-based treatment [17, 18]. The most important molecular mechanism of lithium is the mediatory action on GSK3, which could in turn phosphorylate the transcription factors to turn on the genes related to the cell growth, differentiation, inflammation, etc., such as the Wnt/β-catenin, Myc, and the NF-κB [37–39].

In this study, the influence of the preconditioning on cell proliferation is comparable in different groups (1–10 mmol/L). According to the previous study, the favorable effect of lithium on the proliferation and differentiation of MSC are dose dependent [40]. Satija et al. [41] concluded the promotion on cell proliferation occurred when the concentration was less than 5 mmol/L. It was also supported by de Boer et al. that lower concentration or low activity of Wnt pathway caused the proliferation of uncommitted MSC [42]. Our data is partial consistent with these findings that the cells treated with 4 mmol/L LiCl exhibited the highest proliferation rate in normal condition. Though the inhibition on GSK-3b would cause the activation of Wnt pathway, the preconditioning treatment of LiCl here (1–10 mmol/L) did not cause significant reduction of GSK-3b, nor during the NP differention process.

Besides, our data first proved that the preconditioning of lithium enhanced the adaptation of ADSC to the degenerative IVD-like condition, including a reduced cell death and an increased ECM deposit. And our results also suggested the participant of the elevated cellular ROS in the certain process during preconditioning. Previous studies has found that ROS plays important roles in cell fate decision and that an optimal ROS is critical for nuclear reprogramming and the generation of pluripotent stem cells [43]. Subsequently, the elevated ROS after LiCl preconditioning caused an activation of ERK1/2, which had been found critical during the adaptation of transplanted stem cell to harsh IVD condition [11], and also to play key role in anti-inflammation response [44]. Consistently, the addition of antioxidant to prevent the ROS generation during preconditioning attenuated the enhancement on the cellular adaptation to harsh condition. Therefore, we considered the critical role of ROS/ERK axis on the preconditioning effects of ADSC in this study.

Besides, the preconditioning with LiCl has also been reported with strong protection to MSC against apoptosis by upregulating the gene expression of Naip1, Erc1, and Faim2 [18]. In addition to the anti-apoptosis gene, the lithium activates Akt-1, which is a critical protein kinase that modulate apoptotic pathway [35]. Both mechanisms may be included in the adaptation of preconditioned ADSC against the stressed degenerative condition, and the enhanced ECM deposit in degenerative IVD-like condition. The influence of lithium preconditioning to stem cell could be complex; further study is intended to uncover the interaction between preconditioning with lithium and the cellular adaptation.

## Conclusion

In this study, we found the positive influences of preconditioning with LiCl to ADSC, including the proliferation and NP-like cell differentiation efficacy under normal condition, and the cell adaptation in a hostile degenerative IVD-like condition with a reduced cell death and enhanced ECM deposit, and superior therapeutic effects to IVDD in vivo. And these influences were closely related to the activation of ROS/ERK axis in preconditioned ADSC. This novel inorganic method of LiCl preconditioning exerts promising application potential to boost the ADSC activity for the IVD regeneration.

## Data Availability

The datasets used and/or analyzed during the current study are available from the corresponding author on reasonable request.

## Abbreviations

ADSC: Adipose-derived stem cell
IVD: Intervertebral disc
LDH: Lactate dehydrogenase
LiCl: Lithium chloride
NP: Nucleus pulpous
ECM: Extracellular matrix
ROS: Reactive oxidative species
LBP: Low back pain
IVDD: Intervertebral disc degeneration
Col II: Type II collagen
BMSC: Bone marrow-derived mesenchymal stem cell
GSK: Glycogen synthase kinase
SD: Sprague-Dawley
FBS: Fetal bovine serum
DMEM: Dulbecco’s modified Eagle’s medium
ITS: Insulin-transferrin-selenium
BSA: Bovine serum albumin
SDS-PAGE: Sodium dodecyl sulfate polyacrylamide gel electrophoresis
PVDF: Polyvinylidene difluoride
ACAN: Aggrecan
AF: Annulus fibrosus
H&E: Hematoxylin and eosin
S-O: Safranin O-fast green
MMP13: Matrix metalloproteinase 13
